# Genetically predicted circulating levels of cytokines and the risk of osteoarthritis: A mendelian randomization study

**DOI:** 10.3389/fgene.2023.1131198

**Published:** 2023-03-14

**Authors:** Dalin Su, Yanhong Ai, Guoyong Zhu, Yubiao Yang, Pengyi Ma

**Affiliations:** ^1^ Xiangyang Hospital of Traditional Chinese Medicine, Xiangyang, China; ^2^ Department of Orthopedics, Tianjin Medical University General Hospital, Tianjin, China

**Keywords:** osteoarthritis, mendelian randomization, RANTES, MIP-1 beta, TNF-beta

## Abstract

**Background:** The association between inflammatory cytokines and osteoarthritis (OA) has been reported in several observational studies, but the causal relationship between these two remains unknown. Hence, we performed this two-sample Mendelian randomization (MR) to confirm the causal relationship between circulating levels of inflammatory factors and osteoarthritis risk.

**Method:** We used genetic variants associated with cytokine circulation levels from a meta-analysis of genome-wide association studies (GWASs) in 8,293 Finns as instrumental variables and obtained OA data from the United Kingdom Biobank, including a total of 345,169 subjects of European ancestry (66,031 diagnosed OA cases and 279,138 controls). Inverse variance weighting (IVW), MR-Egger, Wald Ratio, weighted median, and MR multiplicity residual sums with outliers (MR-PRESSO) were used.

**Result:** We found a causal relationship between circulating levels of macrophage inflammatory protein-1beta (MIP-1β) and risk of OA (OR = 0.998, 95% CI = 0.996–0.999*p* = 9.61 × 10^−5^); tumour necrosis factor beta (TNF-β) was also causally associated with risk of OA (OR = 0.996,95%CI = 0.994–0.999, *p* = 0.002); finally we found a suggestive association between C-C motif chemokine ligand 5(CCL5, also called Rantes) and OA risk (OR = 1.013, 95%CI = 1.002–1.024,*p* = 0.016).

**Conclusion:** Our findings offer promising leads for the development of new therapeutic targets in the treatment of osteoarthritis. By identifying the role of inflammatory cytokines in this debilitating condition through a genetic epidemiological approach, our study contributes to a better understanding of the underlying disease mechanisms. These insights may ultimately pave the way for more effective treatments that improve patient outcomes.

## 1 Introduction

Osteoarthritis (OA) is a kind of joint, slow-onset, and highly disabling disease in clinical practice which causes irreversible joint lesions such as bone resorption, bone destruction, and bone fibrosis, eventually leading to disability (Duncan et al., 2009). In recent years, the incidence and disability rates of osteoarthritis have continued to rise, making it the second most common chronic disease among older patients, after hypertension, hyperglycemia, and hyperlipidemia. This trend poses an ongoing threat to public health systems worldwide, highlighting the urgent need for effective prevention and treatment strategies to address this growing health concern (Hawker, 2019). Osteoarthritis of the knee is the primary type of osteoarthritis, ranking 11th in the world in terms of disability and posing a substantial economic burden to patients, families, and society (Losina et al., 2015).

For many years, osteoarthritis was considered a “wear and tear” disease. However, with the development of clinical medicine, it has been recognized as a complex, multifaceted disease (Kraus et al., 2015). According to the International Osteoarthritis Research Society, osteoarthritis is initially manifested as a molecular disorder with joint tissue metabolism abnormalities, then followed by anatomical and/or physiological disturbances characterized by cartilage degradation, bone remodelling, bone redundancy formation, joint inflammation, and loss of normal joint function. There is growing evidence showing that inflammation plays a vital role in the pathogenesis of OA, and the link between inflammatory factors and OA risk has been increasingly reported (Molnar et al., 2021). Previous studies have shown that OA patients have higher circulating levels of several inflammatory cytokines than healthy controls. For example, Jiří Baloun et al. (2020) showed elevated levels of nine inflammatory mediators (e.g., eosinophil chemokine, monocyte chemotactic protein 1, interleukin-10(IL-10), and tumour necrosis factor) in patients with hip osteoarthritis (Baloun et al., 2020). In a meta-analysis designed for 682 individuals, elevated monocyte chemotactic protein-1 (MCP-1) concentrations were observed in patients with OA but not in healthy controls (Ni et al., 2020). It has also been shown that elevated CCL-5 contributes to OA progression (Monibi et al., 2015). However, the relationship between circulating levels of cytokines and the risk of osteoarthritis is questionable due to the limitations of observational studies, such as small sample sizes, follow-up time, and reverse causality, which can mislead the results (Davey Smith and Ebrahim, 2003).

Mendelian randomization approach is well suited to avoid the above problems. At this stage, Mendelian randomization (MR) is one of the most effective methods for making causal inferences. It is becoming increasingly popular as the results of many genome-wide association analyses are published and shared (Bowden et al., 2015). Mendelian randomization methods use genetic information (primarily single nucleotide polymorphisms (SNPs)) as instrumental variables for causal inference, and its studies are based on three hypotheses: 1).

Instrumental variables and exposure factors are strongly correlated; 2) instrumental variables and confounders are not correlated; and 3) instrumental variables are not directly correlated with the outcome, and its effect on the outcome can only be manifested through exposure (Miao et al., 2021). Therefore, MR can effectively avoid the confounding bias of traditional epidemiological studies.

Here, we applied a two-sample MR approach to reveal the causal impact of inflammatory factors on OA risk using pooled statistics of inflammatory cytokines and OA from a genome-wide association study (GWAS) conducted in a large cohort.

## 2 Materials and methods

### 2.1 Data resource

**FIGURE 1:**
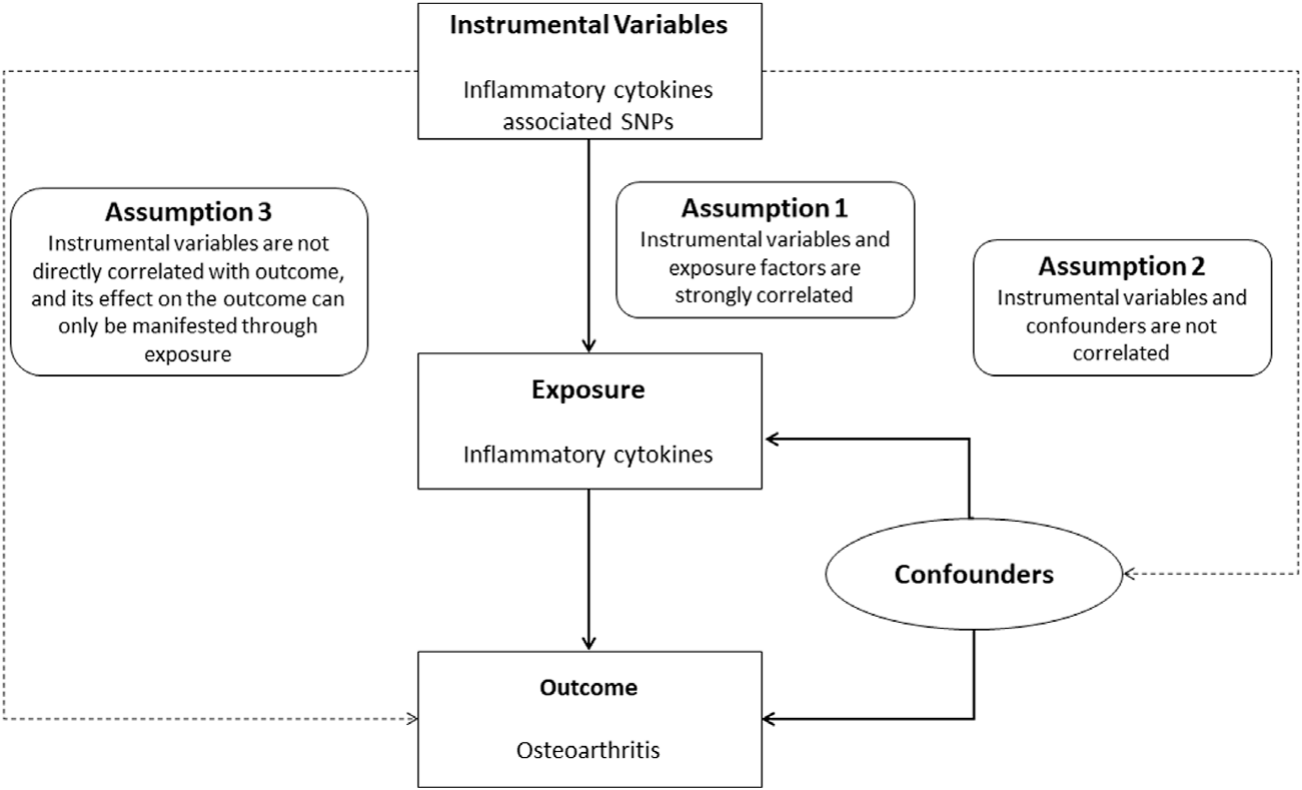
Study flow chart. Schematic representation of two-sample Mendelian randomization analyses for circulating levels of inflammatory cytokines and risk of osteoarthritis disease.

The study design is outlined in Figure 1. For the genetic tool of cytokines, summary statistics were taken from the most comprehensive and extensive cytokine GWAS; The GWAS cytokine meta-analysis included 8,293 Finnish individuals from three separate population-based cohorts: the Young Finns Cardiovascular Risk Study, FINRISK1997 and FINRISK2002 studies (Ahola-Olli et al., 2017). The survey was conducted in Finland, randomly selecting participants between the ages of 25 and 74 from five different geographical areas. The levels of cytokines were measured in the participants’ EDTA plasma, heparin plasma, and serum. Only measurements falling within the detectable range for each cytokine were included in the analysis, and any cytokines with more than 90% of their values missing were excluded (7 out of 48). All participants provided written informed consent.

Regarding the genetic instrument for OA, genetic data for OA were obtained from a large meta-analysis of genome-wide association studies (GWASs) from the United Kingdom Biobank (UKB) (Zengini et al., 2018); genotype data is consistent with 16.5 million variants from the United Kingdom Biobank resource, including 345,169 subjects of European ancestry in total, precisely 66,031 OA cases and 279,138 controls. The clinical diagnosis of OA was based on ICD-10 codes captured from the hospital episode statistic (Zengini et al., 2018). Details of the GWASs included in our study and details of the dataset are summarized in Supplementary Table S1.

### 2.2 Cytokine SNP selection

It is well known, as we mentioned in the previous article, that the MR approach depends on three hypotheses. So, we first selected 5,763 SNPs by genome-wide significance threshold of *p* < 5 × 10^−8^ and false discovery rate (FDR) less than 5% (Annis et al., 2021); then we left 211 SNPs with the lowest *p*-value as independent instruments by linkage disequilibrium (The 1,000 Auton et al., 2015) (LD, *r* ^2^ < 0.1 European 1,000 Genomes Reference Group); we also calculated F-values to assess whether the selected SNPs were strongly associated with exposure (Burgess et al., 2011), and finally calculated that all SNPs had F values well above 10; harmonizing processes were conducted to exclude ambiguous and palindromic SNPs (Supplementary Tables S4, S5). To avoid pleiotropy, we excluded multiple SNPs associated with multiple cytokines, 187 SNPs associated with 23 cytokines were left; Lastly, 176 available SNPs are left in the OA database. (Supplementary Table S2).

### 2.3 Statistical analysis

Since each cytokine has a different number of SNPs, Among cytokines with only one SNP, we selected the Wald ratio as the primary MR analysis (Perry et al., 2021). We selected inverse variance weighting (IVW) as the primary RM analysis for those with two or more SNPs(Lawlor et al., 2008), To assess the potential causative effect of inflammatory factors and the risk of OA. Subsequently, we conducted a Cochrane Q test for IVW to detect heterogeneity. It was observed that most of the results showed no heterogeneity with the *p*-value of more than 0.05. Only a few showed heterogeneity, but our primary MR analysis was IVW; heterogeneity can exist in it, so the presence of heterogeneity in individual results would not have much impact on the prediction of causality (Burgess et al., 2020).

Next, to further assess causality and investigate the presence of pleiotropy, we performed a set of checks, including MR Egger Regression and MR-PRESSO(Cui and Tian, 2021). Additionally, Leave-one-out was used to analyze the possibility that individual SNPs confounded the overall MR analysis. We also used PhenoScanner to examine the potential dimorphic phenotypes in the individual SNPs assessed to eliminate their potential impact on the results.

Sincing including 23 exposure factors, we set the threshold for statistical significance after Bonferroni-correction at 2.17 × 10^−3^ (0.05/23), The *p*-value between the standard threshold (*p* = 0.05) and the statistically significant one after the Bonferroni correction would be taken as suggestive evidence of a potential causal association.

Most of the above work was performed in R analysis software (version 4.0.3), applying to the related R package, including Two sample MR, data array, Etc.

## 3 Results

### 3.1 Causality between MIP-1β and osteoarthritis risks

**FIGURE 2:**
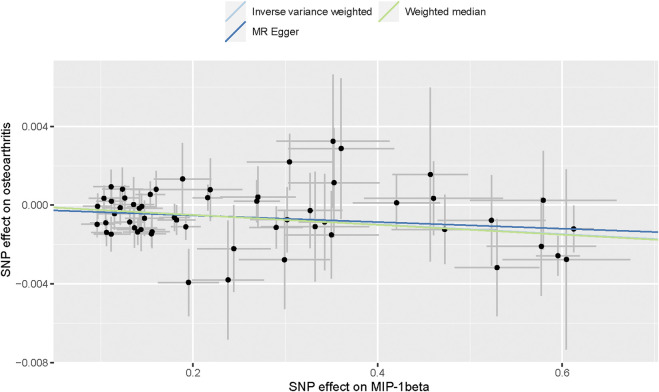
Scatter plot of genetic associations with MIP-1β against osteoarthritis using different MR methods.

**FIGURE 3:**
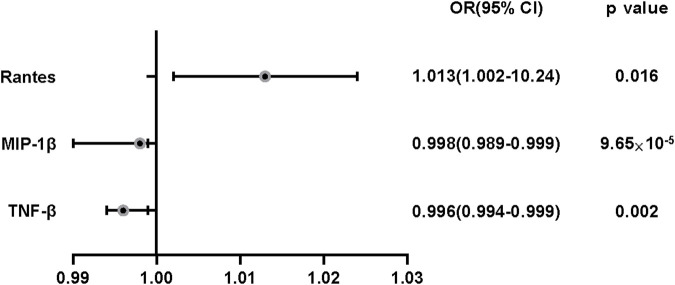
The effect of genetically predicted MIP-1β,TNF-β and Rantes on osteoarthritis risk. CI: confidence interval; OR: odds ratio. OR:odds ratio.

A flow chart of the full-text logic is provided in Figure 1. As shown in Table 1, we set the significance threshold at 2.17 × 10^−3^; we found that high levels of genetically predicted circulating MIP-1β were associated with a reduced risk of OA in the IVW approach (OR = 0.998, 95% CI = 0.996–0.999, *p* = 9.61 × 10^−5^, per 1 Standard deviation (SD) increase.) **(** Figure 2; Figure 3). Furthermore, we did not find heterogeneity using Cochran’s Q test (*p* = 0.45). We also did not find any directional pleiotropy (MR egger-intercept = −0.0001, P for MR egger-intercept = 0.451; P for MR PRESSO global test = 0.739). The results of MR PRESSO and IVW are relatively similar (OR = 0.998,95%CI=(0.996–0.999),*p* = 0.0001.) (Table 1). In addition, two SNPs (rs55771110, rs2049300) were associated with other phenotypes at the genome-wide level (Supplementary Table S3), which we removed at an early stage and had no effect on the results. In the leave-one-out sensitivity analysis, the removal of one SNP did not materially alter the results.

**TABLE 1:** MR estmate, heterogeneity and pleiotropy analysis of all inflammation cytokines. SNP, single-nucleotide polymorphism; OR, odds ratio; CI, confidence interval; MR-PRESSO, Mendelian randomization pleiotropy residual sum and outlier.

Cytokine	NO.of SNPs	OR (95%CI)	P	Hetergeneity test (p)	MR-Egger (intercept,P)	P for MR-PRESSO global test
β-NGF						
Wald ratio	1	1.003 (0.996–1.012)	0.346			
**CTACK**						
Inverse variance weighted	2	1.002 (0.997,1.008)	0.425	0.130		
**Eotaxin**						
Inverse variance weighted	3	1.002 (0.998,1.006)	0.324	0.877		
MR egger	3	1.002 (0.991,1.012)	0.761		4.474 × 10^−6^,0.997	
Weight median	3	1.002 (0.997,1.006)	0.367			
**GRO-α**						
Inverse variance weighted	6	1.003 (0.999,1.005)	0.0668	0.193		
MR egger	6	0.996 (0.989,1.006)	0.262		0.002,0.082	
Weight median	6	1.001 (0.998,1.006)	0.385			
MR-PRESSO	6	1.002 (0.997,1.005)	0.126			0.268
**HGF**						
Inverse variance weighted	2	0.997 (0.990,1.004)	0.367	0.567		
**IL-2rα**						
Inverse variance weighted	2	1.001 (0.997,1.005)	0.526	0.169		
**IL-7**	1					
Wald ratio		0.999 (0.996,1.004)	0.888			
**IL-10**						
Inverse variance weighted	2	1.000 (0.997,1.004)	0.902	0.908		
**IL-12p70**						
Inverse variance weighted	11	0.999 (0.996,1.001)	0.358	0.786		
MR egger	11	1.002 (0.997,1.007)	0.490		−7 × 10^-4^,0.227	
Weight median	11	0.999 (0.996,1.002)	0.623			
MR-PRESSO	11	1.003 (0.997,1.001)	0.275			0.793
**IL-13**						
Inverse variance weighted	4	1.000 (0.997,1.002)	0.726	0.906		
MR egger	4	1.000 (0.995,1.006)	0.701		−1 × 10^-4^,0.842	
Weight median	4	1.000 (0.992,1.007)	0.842			
MR-PRESSO	4	1.000 (0.993,1.005)	0.477			0.943
**IL-16**						
Wald ratio	1	0.999 (0.996,1.002)	0.708			
**IL-18**						
Inverse variance weighted	7	1.002 (0.999,1.005)	0.231	0.333		
MR egger	7	1.006 (0.999,1.014)	0.151		−1 × 10^-3^,0.257	
Weight median	7	1.002 (0.998,1.004)	0.233			
MR-PRESSO	7	1.003 (0.997,1.006)	0.276			0.402
**IP-10**						
Inverse variance weighted	2	0.998 (0.992,1.005)	0.616	0.739		
**MCP-1**						
Inverse variance weighted	6	1.001 (0.995,1.007)	0.744	0.167		
MR egger	6	0.995 (0.980,1.002)	0.500		1 × 10^−3^,0.389	
Weight median	6	1.000 (0.995,1.007)	0.762			
MR-PRESSO	6	0.997 (0.994,1.005)	0.757			0.215
**MIG**						
Wald ratio	1	0.998 (0.989,1.006)	0.579			
**MIP-1β**						
Inverse variance weighted	57	0.998 (0.996,0.999)	9.641 × 10^−5^	0.734		
MR egger	57	0.998 (0.996,1.000)	1.678 × 10^−1^		−1 × 10^-4^,0.450	
Weight median	57	0.997 (0.996,0.999)	1.020 × 10^−2^			
MR-PRESSO	57	0.998 (0.996,0.999)	0.0001			0.739
**PDGF-bb**						
Inverse variance weighted	9	1.001 (0.999,1.005)	0.268	0.671		
MR egger	9	1.005 (0.999,1.012)	0.171		−7 × 10^-4^,0.291	
Weight median	9	1.001 (0.997,1.005)	0.508			
MR-PRESSO	9	1.002 (0.998,1.005)	0.229			0.705
**RANTES**						
Wald ratio	1	1.013 (1.002,1.024)	0.016			
**SCF**						
Inverse variance weighted	2	0.992 (0.982,1.002)	0.131	0.227		
**SCGF-β**						
Inverse variance weighted	7	0.998 (0.993,1.002)	0.405	0.011		
MR egger	7	0.998 (0.988,1.008)	0.695		2.60 × 10^−5,^0.987	
Weight median	7	0.999 (0.995,1.003)	0.594			
MR-PRESSO	7	0.998 (0.993,1.003)	0.437			0.022
**TNF-β**						
Inverse variance weighted	3	0.996 (0.994,0.999)	0.002	0.343		
MR egger	3	0.989 (0.980,0.999)	0.285		7 × 10^−3^,0.403	
Weight median	3	0.996 (0.993,0.998)	0.001			
**TRAIL**						
Inverse variance weighted	22	1.003 (0.998,1.002)	0.720	0.149		
MR egger	22	1.000 (0.997,1.002)	0.779		3 × 10^−4^,0.479	
Weight median	22	1.001 (0.998,1.004)	0.302			
MR-PRESSO	22	1.000 (0.998,1.002)	0.724			0.135
**VEGF**						
Inverse variance weighted	20	0.999 (0.997,1.000)	0.276	0.734		
MR egger	20	1.001 (0.997,1.004)	0.573		−4 × 10^-4^,0.210	
Weight median	20	1.000 (0.998,1.002)	0.839			
MR-PRESSO	20	1.001 (0.998,1.004)	0.233			0.694

### 3.2 Causality between TNF-β and osteoarthritis risks

**FIGURE 4:**
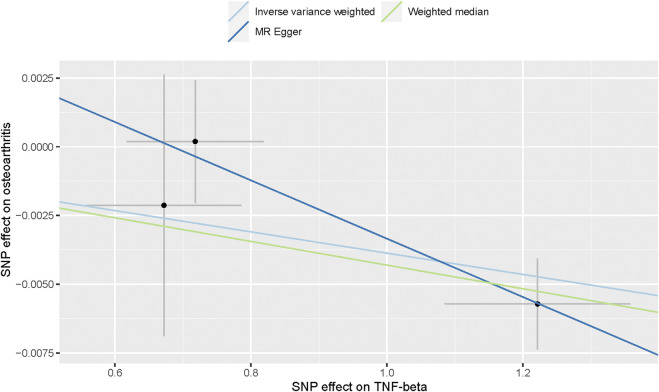
Scatter plot of genetic associations with TNF-α against osteoarthritis using different MR methods.

Also, we found by IVW analysis that increased circulating levels of TNF-β were also associated with a reduced risk of OA (OR = 0.996, 95% CI = 0.994–0.999, *p* = 0.002, per 1 Standard deviation (SD) increase.) (Figure 4; Figure 3), and did not show heterogeneity (Cochrane Q test, *p* = 0.343); nor did it show directional pleiotropy (MR egger-intercept = 0.007, p for MR egger-intercept = 0.403) (Table 1).

### 3.2 Causality between rantes and osteoarthritis risks

In addition, we identified Rantes (Table 1; Figure 3), for which we found a suggestive association between circulating levels and OA risk in Wald ratio analysis, with a 1.3% increase in the risk of OA for each increase in SD (OR = 1.013, 95% CI = 1.002–1.024, *p* = 0.016.); according to PhenoScanner, we did not find the SNP correlated with other phenotypes, suggesting that it does not contribute to the risk of OA through the non-exposure route.

Apart from TNF-β, MIP-1β and Rantes, the other 20 cytokines (e.g., VEGF, GRO-α, Trail, MIG, IL-7, IL -17) did not show any association with the risk of OA in either IVW primary MR analysis or in other secondary analyses (Table 1). In the heterogeneity assay, most of the cytokines were significantly non-heterogeneous, except for SCGF-β (*p* = 0.011). MR-egger regression did not show pleiotropy in *p* values for all cytokines. An additional solidity test, the MR-PRESSO assay, did not show any abnormal values except SCGF-β (*p* = 0.022).

## 4 Discussion

Osteoarthritis (OA), characterized by cartilage lesions, is a chronic degenerative disorder with a high prevalence and disability rate (Glyn-Jones et al., 2015). With the development of clinical medicine, OA has changed from a superficial cartilage “wear and tear” disease to a diverse disease with complex pathogenesis affecting all tissues in the joint (Primorac et al., 2020). OA has seriously affected the quality of life, especially for the elderly, and imposed a severe economic burden on families and the country. Still, the biological mechanisms underlying OA etiology are poorly understood. Due to these limitations, we performed a two-sample MR analysis; our study is the first MR analysis to determine whether inflammatory cytokine levels are associated with OA risk based on genetic data from publicly available databases.

We obtained evidence showing a causal association between high levels of MIP-1β/TNF-β and decreased risk of OA; increased circulating levels in Rantes had a suggestive association with increased risk OA.

Macrophage inflammatory protein-1beta (MIP-1β/CCL4) is the essential chemotactic cytokine in the immune response against infection and inflammation; A chemokine (chemotactic factors) is a small cytokine that attracts other cells to a local area to exert its biological effects. Chemokines, which can be secreted by leukocytes and some tissue cells, are a protein family with over 60 members, mostly 8–10 kDa in molecular weight (Allen et al., 2007). Few clinical observational studies or Meta-analyses have been performed correlation between MIP-1β and OA, but our current MR analysis determined that high levels of circulating MIP-1b were associated with a reduced risk of OA (OR = 0.998, 95% CI = 0.996–0.999, *p* = 9.61 × 10^−5^, per 1 Standard deviation (SD) increase).

In addition, our MR analysis revealed a potential association between the chemokine CCL5, also known as Rantes, and an increased risk of osteoarthritis (OA). CCL5 is a member of the CC chemokine family and has been shown to regulate the expression and secretion of normal or activated T Cells. Our findings suggest that increased circulating levels of CCL5 may contribute to the development of OA (Suffee et al., 2011). Several observational studies have previously demonstrated a link between CCL5 and OA; Beekhuizen et al. reported that the most significantly elevated mediator in synovial fluid in the articular cavity of patients with OA compared with controls was CCL5. A further study confirmed this, documenting significantly higher CCL5 levels in 18 patients compared with the control group (Beekhuizen et al., 2013). Nevertheless, due to the limitations of studies, the results are suspicious and ambiguous; for the following reasons: small sample size and potential confounders. Our MR analysis, therefore, yielded a more reliable suggestive association; an increase of one SD was associated with a 1.3% increased risk of OA (OR = 1.013, 95% CI = 1.002–1.024, *p* = 0.016).

Biologically, CLL4 is the blocker of infection and inflammation, leading to a better amelioration of various inflammatory conditions through the corresponding immune response. CCL4 and CCL5 are CCR5 receptor-binding ligands, but CCL4 plays a minimal role for CCR5 compared to CCL5 (Molnar et al., 2021). Elevated CCL4 activates the NF-kappaB signalling pathway, which leads to the development of OA inflammation is effectively mitigated. Several studies have elucidated the relationship between NF-kappaB and OA. NF-kappaB is strongly associated with chondrocyte survival and apoptosis in osteoarthritis (Barreto et al., 2020). In a study by Tianlong Pan et al., it has been shown that activation of the NF-kappaB signalling pathway inhibited chondrocyte apoptosis induced by inflammatory factors (especially interleukin-1β), thereby delaying the progression of OA (Pan et al., 2017). CCL5 and CCL4 have opposite effects, with CCL5 binding to CCR5 leading to cartilage breakdown in the joint (Borzì et al., 2000). The main reason for this is that CCL5 and CCR5 binding to each other regulates the expression of MMP-3, which leads to the inhibition of proteoglycan synthesis and increases the release of proteoglycan, thereby degrading cartilage (Masuko-Hongo and Yudoh, 2005). CCL5/CCR5 not only degrades cartilage but also increases the production of the remaining inflammatory mediators (such as IL6) by human synovial fibroblasts through the PKCδ/c-Src/c-Jun signal pathway and AP-1 signaling pathways, allowing further development of OA (Tang et al., 2010).

Tumour necrosis factor beta (TNF-β), also called lymphotoxin A, is a close homolog of tumour necrosis factor alpha (TNF-α) (Aggarwal et al., 2012). In the present MR analysis, we found that elevated circulating levels of TNF-β were associated with a decreased risk of developing OA (OR = 0.996,95% CI = 0.994–0.999, *p* = 0.002, per 1 Standard deviation (sd) increase). TNF-β, as well as TNF-α, could bind to TNF receptors (TNFR 1 and TNFR2) (Doss C G, 2014). Prior studies have suggested that the sequences of TNF-β and TNF-α are similar, so that TNF-β increases the risk of inflammatory diseases as does the TNF-α. However, the affinity of TNF-β for receptors is much lower than that of TNF-α, and TNF-β is 1,000 times less effective in receptor activation. Therefore, higher levels of TNF-β can compete with TNF-α for the TNFR1 and TNFR2 receptor sites, and act as an antagonist of TNF-α, reducing the risk of OA.

Our study has several strengths. (1) This is the first MR study to elucidate the relationship between inflammatory cytokines and OA risk. (2) Unlike observational studies, our current study avoids confounders and reverse causality to the greatest extent possible, providing a reliable pair of cause-and-effect relationships. (3) Our study data were obtained from the publicly available GWASS database with a large amount of original study data, which provided a strong guarantee for this study. (4) Unlike the time-consuming randomized controlled studies (RCTs), the cost of time and money invested in this study is highly cost-effective for the results we obtained. However, this study also has some limitations. First, because the data in the database were sourced from Europe, the study was limited to European participants, and the usefulness of the results for other populations remains to be seen. (润色) Second, this study ignored the diversity of OA diseases and did not analyze site-specific OA, and cytokines may have a causal effect on knee OA/hip OA. Third, for the 20 cytokines that did not yield significance in the current study, a relationship between them and OA cannot be excluded, possibly due to the small number of SNPs involved in the study and the lack of MR computational power. Fourth, cytokines are a dynamic indicator, and unlike other indicators such as weight, MR does not address the dynamics of cytokine levels.

## 5 Conclusion

Our study finally identified a causal association between circulating levels of TNF-β, MIP-1β and OA risk and a suggestive link between Rantes and OA. Our study may provide a deeper understanding of the pathogenesis of OA, as well as the development of effective management strategies for the clinic. We suggest that TNF-β, MIP-1β, and Rantes may serve as potential therapeutic targets for OA development.

## Data Availability

The original contributions presented in the study are included in the article/Supplementary Material, further inquiries can be directed to the corresponding authors.

## References

[B1] Aggarwal B. B. Gupta S. C. Kim J. H. (2012). Historical perspectives on tumor necrosis factor and its superfamily: 25 years later, a golden journey. Blood 119, 651–665. 10.1182/blood-2011-04-325225 22053109 PMC3265196

[B2] Ahola-Olli A. V. Würtz P. Havulinna A. S. Aalto K. Pitkänen N. Lehtimäki T. (2017). Genome-wide association study identifies 27 loci influencing concentrations of circulating cytokines and growth factors. Am. J. Hum. Genet. 100, 40–50. 10.1016/j.ajhg.2016.11.007 27989323 PMC5223028

[B3] Allen S. J. Crown S. E. Handel T. M. (2007). Chemokine:Receptor structure, interactions, and antagonism. Annu. Rev. Immunol. 25, 787–820. 10.1146/annurev.immunol.24.021605.090529 17291188

[B4] Annis A. Pandit A. LeFaive J. Taliun S. G. Fritsche L. VandeHaar P. (2021). False discovery rates for genome-wide association tests in biobanks with thousands of phenotypes. Res. Square, 10.21203/rs.3.rs-873449/v1

[B12] Auton A. Brooks L. D. Durbin R. M. Garrison E. P. Kang H. M. Korbel J. O. (2015). A global reference for human genetic variation. Nature 526, 68–74. 10.1038/nature15393 26432245 PMC4750478

[B5] Baloun J. Kropáčková T. Hulejová H. Tomčík M. Růžičková O. Šléglová O. (2020). Chemokine and cytokine profiles in patients with hand osteoarthritis. Biomolecules 11, 4. 10.3390/biom11010004 33375165 PMC7822191

[B6] Barreto G. Manninen M. Eklund K. K. (2020). Osteoarthritis and toll-like receptors: When innate immunity meets chondrocyte apoptosis. Biology 9, 65. 10.3390/biology9040065 32235418 PMC7235883

[B7] Beekhuizen M. Gierman L. M. van Spil W. E. Van Osch G. J. V. M. Huizinga T. W. J. Saris D. B. F. (2013). An explorative study comparing levels of soluble mediators in control and osteoarthritic synovial fluid. Osteoarthr. Cartil. 21, 918–922. 10.1016/j.joca.2013.04.002 23598178

[B8] Borzì R. M. Mazzetti I. Cattini L. Uguccioni M. Baggiolini M. Facchini A. (2000). Human chondrocytes express functional chemokine receptors and release matrix-degrading enzymes in response to C-X-C and C-C chemokines. Arthritis Rheum. 43, 1734–1741. 10.1002/1529-0131(200008)43:8<1734::AID-ANR9>3.0.CO;2-B 10943863

[B9] Bowden J. Davey Smith G. Burgess S. (2015). Mendelian randomization with invalid instruments: Effect estimation and bias detection through egger regression. Int. J. Epidemiol. 44, 512–525. 10.1093/ije/dyv080 26050253 PMC4469799

[B10] Burgess S. Davey Smith G. Davies N. M. Dudbridge F. Gill D. Glymour M. M. (2020). Guidelines for performing Mendelian randomization investigations. Wellcome Open Res. 4, 186. 10.12688/wellcomeopenres.15555.2 32760811 PMC7384151

[B11] Burgess S. Thompson S. G. CRP CHD Genetics Collaboration (2011). Avoiding bias from weak instruments in Mendelian randomization studies. Int. J. Epidemiol. 40, 755–764. 10.1093/ije/dyr036 21414999

[B13] Cui Z. Tian Y. (2021). Using genetic variants to evaluate the causal effect of serum vitamin D concentration on COVID-19 susceptibility, severity and hospitalization traits: A mendelian randomization study. J. Transl. Med. 19, 300. 10.1186/s12967-021-02973-5 34246301 PMC8271325

[B14] Davey Smith G. Ebrahim S. (2003). Mendelian randomization’: Can genetic epidemiology contribute to understanding environmental determinants of disease? Int. J. Epidemiol. 32, 1–22. 10.1093/ije/dyg070 12689998

[B15] Doss C. G, P. Agoramoorthy G. Chakraborty C. (2014). TNF/TNFR: Drug target for autoimmune diseases and immune-mediated inflammatory diseases. Front. Biosci. 19, 1028–1040. 10.2741/4265 24896334

[B16] Duncan R. Peat G. Thomas E. Wood L. Hay E. Croft P. (2009). Does isolated patellofemoral osteoarthritis matter? Osteoarthr. Cartil. 17, 1151–1155. 10.1016/j.joca.2009.03.016 19401244

[B17] Glyn-Jones S. Palmer A. J. R. Agricola R. Price A. J. Vincent T. L. Weinans H. (2015). Osteoarthritis. Lancet 386, 376–387. 10.1016/S0140-6736(14)60802-3 25748615

[B18] Hawker G. A. (2019). Osteoarthritis is a serious disease. Clin. Exp. Rheumatol. 37 Suppl 120, 3–6. 31621562

[B19] Kraus V. B. Blanco F. J. Englund M. Karsdal M. A. Lohmander L. S. (2015). Call for standardized definitions of osteoarthritis and risk stratification for clinical trials and clinical use. Osteoarthr. Cartil. 23, 1233–1241. 10.1016/j.joca.2015.03.036 PMC4516635 25865392

[B20] Lawlor D. A. Harbord R. M. Sterne J. A. C. Timpson N. Davey Smith G. (2008). Mendelian randomization: Using genes as instruments for making causal inferences in epidemiology. Stat. Med. 27, 1133–1163. 10.1002/sim.3034 17886233

[B21] Losina E. Paltiel A. D. Weinstein A. M. Yelin E. Hunter D. J. Chen S. P. (2015). Lifetime medical costs of knee osteoarthritis management in the United States: Impact of extending indications for total knee arthroplasty. Arthritis Care Res. 67, 203–215. 10.1002/acr.22412 PMC4422214 25048053

[B22] Masuko-Hongo K. Yudoh K. (2005). The role of inflammatory mediators in cartilage degradation. Curr. Rheumatol. Rev. 1, 119–124. 10.2174/1573397054023164

[B23] Miao L. Deng G.-X. Yin R.-X. Nie R.-J. Yang S. Wang Y. (2021). No causal effects of plasma homocysteine levels on the risk of coronary heart disease or acute myocardial infarction: A mendelian randomization study. Eur. J. Prev. Cardiol. 28, 227–234. 10.1177/2047487319894679 33838042

[B24] Molnar V. Matišić V. Kodvanj I. Bjelica R. Jeleč Ž. Hudetz D. (2021). Cytokines and chemokines involved in osteoarthritis pathogenesis. Int. J. Mol. Sci. 22, 9208. 10.3390/ijms22179208 34502117 PMC8431625

[B25] Monibi F. Roller B. Stoker A. Garner B. Bal S. Cook J. (2015). Identification of synovial fluid biomarkers for knee osteoarthritis and correlation with radiographic assessment. J. Knee Surg. 29, 242–247. 10.1055/s-0035-1549022 25927354

[B26] Ni F. Zhang Y. Peng X. Li J. (2020). Correlation between osteoarthritis and monocyte chemotactic protein-1 expression: A meta-analysis. J. Orthop. Surg. 15, 516. 10.1186/s13018-020-02045-2 PMC7654153 33168099

[B27] Pan T. Chen R. Wu D. Cai N. Shi X. Li B. (2017). Alpha-Mangostin suppresses interleukin-1β-induced apoptosis in rat chondrocytes by inhibiting the NF-κB signaling pathway and delays the progression of osteoarthritis in a rat model. Int. Immunopharmacol. 52, 156–162. 10.1016/j.intimp.2017.08.021 28915439

[B28] Perry B. I. Burgess S. Jones H. J. Zammit S. Upthegrove R. Mason A. M. (2021). The potential shared role of inflammation in insulin resistance and schizophrenia: A bidirectional two-sample mendelian randomization study. PLOS Med. 18, e1003455. 10.1371/journal.pmed.1003455 33711016 PMC7954314

[B29] Primorac D. Molnar V. Rod E. Jeleč Ž. Čukelj F. Matišić V. (2020). Knee osteoarthritis: A review of pathogenesis and state-of-the-art non-operative therapeutic considerations. Genes 11, 854. 10.3390/genes11080854 32722615 PMC7464436

[B30] Suffee N. Richard B. Hlawaty H. Oudar O. Charnaux N. Sutton A. (2011). Angiogenic properties of the chemokine RANTES/CCL5. Biochem. Soc. Trans. 39, 1649–1653. 10.1042/BST20110651 22103502

[B31] Tang C.-H. Hsu C.-J. Fong Y.-C. (2010). The CCL5/CCR5 axis promotes interleukin-6 production in human synovial fibroblasts: CCL5 induces IL-6 production. Arthritis Rheum. 62, 3615–3624. 10.1002/art.27755 20862675

[B32] Zengini E. Hatzikotoulas K. Tachmazidou I. Steinberg J. Hartwig F. P. Southam L. (2018). Genome-wide analyses using UK Biobank data provide insights into the genetic architecture of osteoarthritis. Nat. Genet. 50, 549–558. 10.1038/s41588-018-0079-y 29559693 PMC5896734

