# Supplementary material for: Genetically predicted circulating levels of cytokines and the risk of osteoarthritis: A mendelian randomization study
Source: Front Genet. 2023 Mar 14;14:1131198. doi: 10.3389/fgene.2023.1131198 (PMC10043178; doi:10.3389/fgene.2023.1131198)
Supplement: Supplementary file 1 [file DataSheet1.docx]

| Exposures/Outcomes | Study/consortium | Participants | Available website |
| --- | --- | --- | --- |
| Cytokines | YFS, FINRISK1997, FINRISK2002 | 8,293 Finns | https://www.finngen.fi/fi |
| Osteoarthritis | UKB | 456,348 unrelated European-descent individuals | http://www.nealelab.is/uk-biobank |

Table S1：Detailed information of the studies and datasets used in the present study.

YFS：The Cardiovascular Risk in Young Finns Study

Table S2

**Genetic instruments of cytokines included in the present study.**

| **Cytokines** | **Abbreviation** | **Total N** | **SNPs（N）** |
| --- | --- | --- | --- |
| Beta nerve growth factor | β-NGF | 3531 | 1 |
| Cutaneous T-cell attracting (CCL27) | CTACK | 3631 | 2 |
| Eotaxin (CCL11) | Eotaxin | 8153 | 3 |
| Basic fibroblast growth factor | FGF-basic | 7565 | 0 |
| Granulocyte colony-stimulating factor | G-CSF | 7904 | 0 |
| Growth regulated oncogene-α (CXCL1) | GRO-α | 3505 | 6 |
| Hepatocyte growth factor | HGF | 8292 | 2 |
| Interleukin-1 receptor antagonist | IL-1rα | 3638 | 0 |
| Interleukin-1-beta | IL-1β | 3309 | 0 |
| Interleukin-2 | IL-2 | 3475 | 0 |
| Interleukin-2 receptor, alpha subunit | IL-2rα | 3677 | 2 |
| Interleukin-4 | IL-4 | 8124 | 0 |
| Interleukin-5 | IL-5 | 3364 | 0 |
| Interleukin-6 | IL-6 | 8189 | 0 |
| Interleukin-7 | IL-7 | 3409 | 1 |
| Interleukin-8 (CXCL8) | IL-8 | 3526 | 0 |
| Interleukin-9 | IL-9 | 3634 | 0 |
| Interleukin-10 | IL-10 | 3685 | 2 |
| Interleukin-12p70 | IL-12p70 | 8270 | 11 |
| Interleukin-13 | IL-13 | 3557 | 4 |
| Interleukin-16 | IL-16 | 3483 | 1 |
| Interleukin-17 | IL-17 | 7760 | 0 |
| Interleukin-18 | IL-18 | 3636 | 8 |
| Interferon gamma-induced protein 10 (CXCL10) | IP-10 | 3685 | 2 |
| Monocyte chemotactic protein-1 (CCL2) | MCP-1 | 8293 | 9 |
| Macrophage migration inhibitory factor (glycosylation-inhibiting factor) | MIF | 3494 | 0 |
| Monokine induced by interferon-gamma (CXCL9) | MIG | 3682 | 1 |
| Macrophage inflammatory protein-1α (CCL3) | MIP-1α | 3522 | 0 |
| Macrophage inflammatory protein-1β (CCL4) | MIP-1β | 8243 | 57 |
| Platelet derived growth factor BB | PDGF-bb | 8293 | 9 |
| Regulated on activation, normal T Cell expressed and secreted (CCL5) | RANTES | 3421 | 1 |
| Stem cell factor | SCF | 8290 | 2 |
| Stem cell growth factor beta | SCGF-β | 3682 | 7 |
| Stromal cell-derived factor-1 alpha (CXCL12) | SDF-1α | 5998 | 0 |
| Tumor necrosis factor-alpha | TNF-α | 3454 | 0 |
| Tumor necrosis factor-beta | TNF-β | 1559 | 3 |
| TNF-related apoptosis inducing ligand | TRAIL | 8186 | 22 |
| Vascular endothelial growth factor | VEGF | 7118 | 20 |

Abbreviation: SNP, single nucleotide polymorphism.

Table S3：Documented pleiotropic associations for the instrumental variables used for circulating MIP-1beta levels.

| SNP | Traits | P-value | PubMed |
| --- | --- | --- | --- |
| rs2049300 | High light scatter  reticulocyte count | 1.18e-12 | 27863252 |
|  | Reticulocyte count | 1.46e-12 | 27863252 |
|  | Body mass index | 3.59e-06 | UKBB |
| rs55771110 | Monocyte percentage of white  cels | 4.94e-09 | 27863252 |
|  | Mean platelet  volume | 1.67e-06 | 27863252 |
|  | Weight | 6.48e-06 | UKBB |

SNP: single nucleotide polymorphism. UKBB:UK biobank
